# The Acute Post-Activation Performance Enhancement of the Bench Press Throw in Disabled Sitting Volleyball Athletes

**DOI:** 10.3390/ijerph18073818

**Published:** 2021-04-06

**Authors:** Michal Krzysztofik, Patryk Matykiewicz, Diana Celebanska, Jakub Jarosz, Eliza Gawel, Anna Zwierzchowska

**Affiliations:** Institute of Sport Sciences, Jerzy Kukuczka Academy of Physical Education in Katowice, 40-065 Katowice, Poland; p.matykiewicz@awf.katowice.pl (P.M.); d.celebanska@awf.katowice.pl (D.C.); j.jarosz@awf.katowice.pl (J.J.); elizagawel77@gmail.com (E.G.); a.zwierzchowska@awf.katowice.pl (A.Z.)

**Keywords:** power output, upper-body strength, resistance training, paralympic volleyball, explosive training, complex training, velocity-based training

## Abstract

The purpose of the present study was to examine the acute effects of the bench press exercise with predetermined velocity loss percentage on subsequent bench press throw (BPT) performance with raised legs or feet on the floor among disabled, sitting volleyball players. Twelve elite sitting volleyball athletes (age = 33 ± 9 years; body mass = 84.7 ± 14.7 kg; relative bench press maximum strength = 1.0 ± 0.3 kg/body mass) took part in this study. The experiment was performed following a randomized crossover design, where each participant performed a single set of bench press with a 60% one-repetition maximum (1RM) to a 10% decrease of mean bar velocity as a conditioning activity (CA). The BPT with a 60%1RM was performed to assess changes in peak power (PP), peak velocity (PV) before and after the CA. The differences between analyzed variables before and after the CA were verified using two-way repeated-measures ANOVA (condition × time; 2 × 2). The ANOVA showed a significant main effect of time for peak bar velocity (*p* = 0.03; η^2^ = 0.312) and peak power output (*p* = 0.037; η^2^ = 0.294). The post hoc comparison showed a significant increase in post-CA peak bar velocity and peak power for raised legs condition in comparison with pre-CA value (*p* = 0.02, *p* = 0.041, respectively). The present study showed that the subsequent BPT performed with raised legs could be enhanced by the bench press with a 60% 1RM to a 10% mean bar velocity decrease as a CA among disabled sitting volleyball players. Therefore, athletes and coaches can consider performing a bench press throw with raised legs without compromising performance.

## 1. Introduction

Sitting volleyball is an official Paralympic sport. It differs from traditional volleyball mainly in the position of the players, which is determined by the location of contact between the player’s buttocks and the floor on the court. Due to the modified rules of the game (e.g., lower net height), sitting volleyball is even faster than the traditional game [1]. Thus, sitting volleyball is a fast, high-level competitive team sport, demanding power and agility.

Since most sitting volleyball players have lower body disabilities, they need to have a high level of upper-body physical fitness, core muscle strength, and good balance in the sitting position [2]. Besides, they need to make decisions rapidly and move to drop the ball in the opponents’ court. Therefore, the coaches, taking into account these high-speed requirements, to improve the skills of the players, should largely introduce training methods aimed at developing power of the upper body. One such approach includes complex training, the effectiveness of which is based on the postactivation performance enhancement (PAPE) phenomenon.

PAPE has become the focus of many strength and conditioning training programs as it can provide an acute enhancement in strength and power performances as a result of the recent voluntary contractile history [3,4]. In training practice, increased fitness performance is caused by a potentiation complex, which consists of a conditioning activity (CA) (e.g., bench press), followed by a similar movement performed in an explosive manner (e.g., bench press throw) [4,5]. For developing upper body muscle power, the bench press throw exercise is recommended and is considered one of the most effective, since there is a lack of a braking phase at the end, like in bench press, which affects the power output [6,7,8,9]. Furthermore, the bench press throw is associated with overall performance in a variety of sport-specific tasks [9,10]. Therefore, it seems reasonable to use it as a part of potentiation complex as well as testing the ballistic performance of the upper body [11,12].

There are a large number of studies that evaluate the effectiveness of various conditioning activities on the upper-body PAPE effect [5,13,14,15,16,17,18,19,20] in groups of able-bodied athletes, but there is no replication of their protocols on disabled athletes. It seems that there is no reason to believe that this phenomenon may differ between able-bodied and disabled athletes; however, in case of the bench press performed with maximum effort, the position on the bench may have an impact on training outcomes [21]. During the bench press exercise or the BP throw, the athlete has four points of support: head, shoulders and upper back, buttocks, and feet. This positioning ensures a stable position of the body and allows the athlete to achieve a higher level of strength compared to unstable conditions (e.g., balance cushion under the feet) [22]. While in case of able-bodied athletes there is a choice of whether to use stable or unstable conditions underneath the foot when needed (e.g., as an additional stimulus to break through plateaus) [23], in the case of athletes with disabilities of the lower limbs the choice is limited. A prosthesis can help restore the functional performance of an amputee, but for financial reasons, disabled individuals often choose cheap and simple prostheses to provide basic functionality during standing and walking. Thus, the healthy side has to compensate the amputee side to perform daily tasks (in unilateral amputees). Sitting volleyball players often do not have one or both lower limbs, losing or limiting the possibility of using one of the points of support that seems to be the most significant (wide spacing of the feet provides a large base of support). Nevertheless, there is the question of whether sitting volleyball players who have a high level of core muscle strength and balance due to the demands of this discipline still derive any benefits from the point of support provided by the feet.

Taking into account the high-speed requirement of sitting volleyball, and therefore the use of training methods directed at increasing the level of muscular power, this study aimed to assess: (i) differences in the level of power output and bar velocity during the bench press throw with raised legs or supported on the floor and (ii) whether it could be acutely enhanced by the bench press with predetermined velocity loss among disabled, sitting volleyball players. We hypothesized that there would be no differences in the bench press throw performance between raised legs and legs supported on the floor. Moreover, the bench press with predetermined velocity loss will potentiate subsequent bench press throw performance.

## 2. Materials and Methods

### 2.1. Study Design and Procedure

The participants took part in a familiarization and an experimental session within one-week. The first (Mon) session included the determination of the one-repetition maximum (1RM) load in the flat bench press and familiarization with the bench press throw exercise. During the experimental session (Thu), each participant performed a single set of the bench press exercise with 60% 1RM, until mean velocity dropped to 90% of that reached in the CA. To assess changes in peak bar velocity and peak power output, one set of one repetition of the bench press throw was performed on the Smith machine with a load of 30% 1RM either with raised legs or with feet on the floor (in random order), before and after the CA. The participants were instructed to not perform any additional resistance exercises within 72-h of testing to avoid fatigue. Moreover, they were asked to maintain their normal dietary and sleep habits throughout the study and not to use any supplements or stimulants for 24-h before the sessions.

### 2.2. Participants

Twelve elite sitting volleyball athletes (Polish National Team) took part in this study (Table 1). The inclusion criteria were: (a) individuals with a degree of disability, which according to World ParaVolley classified them to compete in sitting volleyball; (b) free from neuromuscular and musculoskeletal disorders; (c) minimum of 2 years of resistance training experience. Seven participants use prostheses, but only for supportive and locomotor activities in everyday life, two athletes with hip exfoliation use orthopedic crutches, one athlete has bilateral amputation of the lower limbs above the knee joints and only uses a wheelchair. The other participants can move without orthopedic support (sagittal defect and muscle atrophy of the lower limbs). All athletes were classified in Paralympic sport according to their current medical status.

The research was carried out during a 5-day training camp of the national team. The study participants were allowed to withdraw from the experiment at any moment. They were informed about the benefits and potential risks of the study before providing their written informed consent for participation. The study protocol was approved by the Bioethics Committee for Scientific Research, at the Academy of Physical Education in Katowice, Poland (No. 9/2012) and performed according to the ethical standards of the Declaration of Helsinki, 2013. To calculate the sample size, statistical software (G*Power, Dusseldorf, Germany) was used. Given the study structure, a 2-way analysis of variance (ANOVA) (2 condition and 2 repeated measures), a small overall effect size (ES) = 0.45, an alpha-error < 0.05, and the desired power (1-ß error) = 0.8, the total sample size resulted in 12 participants.

### 2.3. Familiarization Session and 1RM Strength Test

The participants arrived at the laboratory at the same time of day as the upcoming experimental sessions (in the morning between 9:00 and 11:00 a.m.). Next, they performed a standardized, general warm-up comprised of: work on an ergometer with the upper-body component for 5 min (Keiser M3i Total Body Trainer, Keiser Corporation, Fresno CA, USA) at a resistance approximately of 70 W and cadence within 60–80 rpm; 2 circuits of 10 trunk rotations and side-bends; 10 internal, external and lateral arm swings. After that, the participants performed 15, 10, and 5 BP repetitions using 20%, 40%, and 60% of their estimated 1RM. Hand placement on the barbell was set at 150% individual biacromial distance to ensure consistent hand placement during all experimental sessions. During that evaluation, the participants executed a single repetition with a constant tempo of movement (2 s duration of the eccentric phase and maximum velocity in the concentric phase, with no pause in-between). The loading started at 80% estimated 1RM and if the participant successfully lifted the load, the weight was increased by 2.5 to 10 kg in following attempts until the 1RM for a particular bar condition was obtained. The 1RM was defined as the highest load completed without any help of the spotters. Five-minute rest intervals were allowed between the 1RM attempts, and all 1RM values were obtained within five attempts.

Following the 1RM test, all participants performed one set of a single repetition of the bench press throw on the Smith machine either with raised legs or with the feet supported on the floor in a random order.

### 2.4. Experimental Session

**Figure 1:**
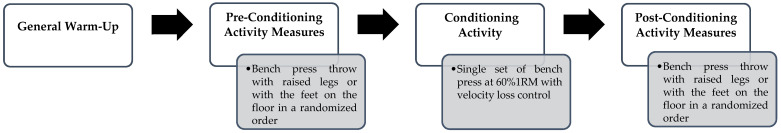
Schematic representation of the experimental session.

During the experimental session, after a standardized warm-up (same as the during familiarization session), the main examinations begin. The participants performed one set of the bench press throw with maximal effort at 30% 1RM on a Smith machine either with raised legs or with the feet supported on the floor in a randomized design as a pre-CA. Each set was performed without bouncing the barbell off the chest, and without intentionally pausing at the transition between the eccentric and concentric phases. After a 5-min rest interval, the participants performed a single set of the bench press exercise as the CA with a 60% 1RM, until mean velocity decreased to 90% of that attained (Figure 1). The effectiveness of this form of CA has been verified by previous study [19]. The participants were asked to execute each repetition with maximal velocity in eccentric and concentric phases. After a 5-min rest interval, the participants repeated the bench press throws as a post-CA. This rest interval time was chosen because earlier studies showed that enhancement of performance after using this type of CA was registered between the 4th and 6th min [15]. To ensure safety and technical proficiency, two strength and conditioning specialists were present during all attempts, and provided spotting for the participants. The bench press throw exercise was chosen since is recommended and considered one of the most effective for developing power output [6,7,8,9]. For achieving accurate measurements and to ensure safety, the Smith machine was used instead of free weights, since it restricts the barbell movement in a vertical direction.

Data were recorded during each bench press throw attempt by a GymAware Powertool linear position transducer (Kinetic Performance Technology, Canberra, Australia), which provides reliable and valid data [24]. The external end of the cable was attached to the side of the bar and provided no resistance. The device was placed on the floor directly under the bar, with the magnetic bottom positioned on a weight plate to ensure no movement during each lift. Velocity and bar movement was recorded at 50 Hz; the load was entered into the software to calculate power and force. Concentric variables were considered for each bench press throw, and included: peak bar velocity (m/s) and peak power output (W). To assess the mean velocity loss during the CA a similar device was used (Tendo Power Analyzer, Tendo Sport Machine, Trencin, Slovakia). A linear position transducer consists of a velocity sensor connected to the bar with a kevlar cable, which, through the interface, immediately transmits the vertical velocity achieved by the barbell to special software installed on the computer. The sampling rate is determined by the velocity of the disk’s rotation. In previous studies, this linear transducer has emerged as a reliable system for measuring barbell velocity and power output during the bench press exercise (intraclass correlation coefficient and coefficient of variation: 0.977 and 9.1% for mean velocity) [25,26].

### 2.5. Statistical Analysis

All statistical analyses were performed using SPSS (version 25.0; SPSS, Inc., Chicago, IL, USA) and were expressed as means with standard deviations (±SD). Statistical significance was set at *p* < 0.05. The normality of data distribution was checked using Shapiro–Wilk tests. Due to the normal distribution of all analyzed data, peak bar velocity and peak power were analyzed with a two-way (condition × time; 2 × 2) ANOVA with repeated measures. Effect sizes for main effects and interaction were estimated by calculating the partial eta squared (η2). Partial eta squared values were classified as small (0.01 to 0.059), moderate (0.06 to 0.137) and large (>0.137). In the event of a significant main effect, post hoc comparisons were conducted using the Bonferroni test. Magnitude of mean differences were expressed with standardized (Cohen) effect sizes; thresholds for qualitative descriptors of Cohen’s d were defined: <0.20 as “trivial”, 0.20–0.49 as “small”, 0.50–0.79 as “moderate”, and >0.80 as “large”. Mauchly’s test of sphericity was conducted to test for the homogeneity of data and if violated (*p* < 0.05), the Greenhouse–Geisser adjustment value was used. The 95% confidence intervals for mean values were also calculated.

## 3. Results

Detailed characteristics of the participants are presented in Table 1. Table 2 contains the differences in performance variables during the bench press throw exercise performed pre- and post-CA. The two-way repeated measures ANOVA indicated no significant condition × time interaction effect for peak bar velocity (*p* = 0.131; η^2^ = 0.166) as well as for peak power output (*p* = 0.096; η^2^ = 0.198). However, there was a significant main effect of time for peak bar velocity (*p* = 0.03; η^2^ = 0.312) and peak power output (*p* = 0.037; η^2^ = 0.294). The post hoc comparison showed a significant increase in post-CA peak bar velocity and peak power for raised legs condition in comparison with pre-CA value (*p* = 0.02, *p* = 0.041; respectively).

## 4. Discussion

The purpose of the present study was to examine if a subsequent bench press throw performed with raised legs or feet supported on the floor could be enhanced by the bench press with predetermined velocity loss as a prior conditioning activity, and whether there would be any differences between these conditions. The primary finding of this study was that the bench press exercises performed with 60% 1RM to a 10% mean bar velocity decrease significantly potentiated subsequent bench press throw performance with raised legs, while no differences in pre- and post-CA values were found when bench press throws were performed with feet on the floor among disabled, sitting volleyball players.

The results of this study partially confirm the initial hypotheses, indicating no differences in pre-CA peak bar velocity and peak power between the bench press throw performed with raised legs or legs supported on the floor in disabled sitting volleyball players. However, a significant performance enhancement after the CA was observed only during the raised legs bench press throw. This result may be related to increased muscle activity of the prime-movers during the bench press (pectoralis major, anterior deltoid, triceps brachii) performed with the raised legs compared to the legs supported on the floor, as indicated by previous studies [27]. This finding was also shown by Golas et al. [21], who found increased involvement of upper body muscles during bench press in an athlete with lower limb disability (limited kinesthetic sense and proprioception). It seems that in such situations the compensation of deficits occurs through the greater activity of the upper body muscles due to the lack of support for the legs. Besides, it cannot be ruled out that it may also be related to the type of prosthesis used by the participants, which may not have provided a sufficient substitute for a healthy extremity and may have even disturbed the support. Nevertheless, it should be emphasized that the sitting volleyball players of this sports discipline have very well-developed core muscle strength, as well as excellent balance in a sitting position [2], which could translate into the obtained differences in the bench press throw performed with raised legs. This finding may be desired by sitting volleyball players, who often complain of low back pain. There is a belief in the fitness community that performing the bench press with raised legs allows reducing the pain in the lumbar region; however, there are no studies to date that would confirm this. Nonetheless, it should be stated that due to the specificity of sport and disability, it is necessary to recognize the importance of strength and power performances in preventing injuries during training and competition. Unilateral leg amputation leads to an asymmetric rotational profile of an athlete with muscular dystonia, which increases the risk of injury. Therefore, there is a particular need to evaluate the external and internal rotator cuff to ensure adequate training in correcting dystonia and preventing trauma. Overall, it seems that athletes and coaches should consider performing the bench press throw with raised legs without compromising performance. Conversely, they can even expect an increase in generated power due to the PAPE effect.

Many studies have been carried out to determine the upper body PAPE effect caused by various CA; however, according to the authors’ knowledge, this is the first to date assessing this phenomenon in the group of disabled athletes. Due to the optimal balance between fatigue and potentiation to achieve performance enhancement induced by CA, the researchers mainly studied how different intensities of the bench press exercises as a CA affect the subsequent bench press throw performance [13,18,28], whereas the recently proposed approach by Tsoukos et al. [19,20] allows for the equalization of fatigue between participants using velocity-based repetition control to induce the same level of fatigue, depending on the strength level and the fatigue characteristics of the muscles involved. Studies by Tsoukos et al. [19,20] have shown that bench press throw performance can be significantly improved by 4.5–7.4% after 4–12 min of bench press performed with light- (40 and 60% 1RM) as well as high-loads (80% 1RM) when mean velocity is not allowed to drop more than 10% of that attained in the first repetition. It is worth noting that in both studies there were considerable differences in the number of performed repetitions to a 10% movement velocity-drop between participants (from two to five repetitions). Hence, the equating of fatigue between participants using velocity-based repetition control allows for the individualization of CA volume and induces the same level of fatigue, depending on the strength level and the fatigue characteristics of the muscles involved. The present study has confirmed the above-mentioned findings on the effectiveness of the use of velocity loss as an individualized method to control the volume of a CA exercise in order to produce subsequent performance enhancement [19,20]. In addition, we found that the PAPE phenomenon can be elicited among disabled athletes and reaches a similar magnitude to that recorded in able-bodied athletes [12].

Considering the high-speed requirement of sitting volleyball, this result may provide important information for coaches and athletes, indicating that they can successfully use the PAPE effect in their training regimens. The monitoring of the drop in movement velocity and thus individualizing the volume load during CA may help strength and conditioning coaches to prescribe effective complex training programs to acutely improve upper body muscle power performance. Additionally, these results justify the introduction of complex training as a new, additional stage of periodization in the development of power output, which opens opportunities for the modification of strength training programs in disabled athletes. Furthermore, when the bench press throw is used in complex training as a post-CA exercise, the results of this study indicated that it should be performed with raised legs in the case of athletes with lower limb disabilities.

Our study has several limitations that should be acknowledged. First, we analyzed only a single CA protocol’s effectiveness to induce the PAPE effect. Therefore, future studies using different CA exercises with variations in intensities (with lower or higher loads), velocity thresholds (especially with greater velocity decreases), and rest-intervals could help optimize PAPE responses. Besides, investigations on the effectiveness of long-term training regimens using the PAPE effect to induce chronic adaptation changes are needed. Second, we enrolled male sitting volleyball elite athletes; thus, the results of this study may not translate to alternative samples. Further studies should make comparisons with able-bodied or disabled athletes, e.g., with swimmers or Paralympic hand-cyclists. Such studies will provide additional information on the protocols for eliciting the PAPE which may require differentiation between athletes and whether the obtained performance improvement reaches a similar magnitude. Finally, we did not perform any physiological, biomechanical, or electromyographical measurements. This may provide insight into the underlying neurophysiological mechanisms. Especially, the use of electromyography would enable us to see the relationships between CA exercise and the activation of particular muscles during subsequent activity.

## 5. Conclusions

In summary, the present study showed that a subsequent bench press throw performed with raised legs could be enhanced by the bench press exercise with a 60% 1RM to a 10% mean bar velocity decrease as a prior conditioning activity among disabled sitting volleyball players. Therefore, athletes and coaches can consider performing the bench press throw with raised legs without compromising performance.

## Figures and Tables

**Table 1:** Descriptive characteristics of study participants.

Characteristic of Group	Mean ± SD
Age (years)	33 ± 9
Body Mass (kg)	84.7 ± 14.7
Body Height * (cm)	185 ± 8
Experience in Paravoleyball (years)	10 ± 9
Experience in RT (years)	14 ± 9
Relative Bench Press 1RM (kg/b.m.)	1 ± 0.3

* excluded bilateral amputation; RT—resistance training; 1RM—one repetition maximum; b.m.—body mass.

**Table 2:** Differences in peak velocity and peak power output during bench press with legs raised and on the floor.

Condition	Pre-CA (95%CI)	Post-CA (95%CI)	ES	Time	Condition	Interaction
Peak Bar Velocity [m/s]						
Raised Legs	1.94 ± 0.19 (1.83–2.05)	1.99 ± 0.22 * (1.86–2.12)	0.24	0.03	0.750	0.131
Legs on the Floor	1.95 ± 0.18 (1.85–2.05)	1.97 ± 0.19 (1.87–2.06)	0.11			
Peak Power [W]						
Raised Legs	583 ± 107 (521–645)	651 ± 172 * (552–750)	0.48	0.037	0.807	0.096
Legs on the Floor	606 ± 117 (539–673)	618 ± 104 (557–678)	0.11			

CA—conditioning activity; CI—confidence interval; ES—effect size; * a significant difference between pre- and post-CA values *p* < 0.05.

## Data Availability

The data presented in this study are available on request from the corresponding author.
